# Atmospheric methane and nitrous oxide: challenges alongthe path to Net Zero

**DOI:** 10.1098/rsta.2020.0457

**Published:** 2021-11-15

**Authors:** Euan G. Nisbet, Edward J. Dlugokencky, Rebecca E. Fisher, James L. France, David Lowry, Martin R. Manning, Sylvia E. Michel, Nicola J. Warwick

**Affiliations:** ^1^ Department of Earth Sciences, Royal Holloway, University of London, Egham TW20 0EX, UK; ^2^ US National Oceanic and Atmospheric Administration, Global Monitoring Laboratory, 325 Broadway, Boulder, CO 80305, USA; ^3^ New Zealand Climate Change Research Institute, School of Geography Environment and Earth Studies, Victoria University of Wellington, Wellington, New Zealand; ^4^ Institute of Arctic and Antarctic Research, Univ. of Colorado, Boulder, CO 80309-0450, USA; ^5^ NCAS, Department of Chemistry, University of Cambridge, Lensfield Road, Cambridge CB2 1EW, UK; ^6^ British Antarctic Survey, Natural Environment Research Council, Cambridge CB3 0ET, UK

**Keywords:** atmospheric methane growth, nitrous oxide growth

## Abstract

The causes of methane's renewed rise since 2007, accelerated growth from 2014 and record rise in 2020, concurrent with an isotopic shift to values more depleted in ^13^C, remain poorly understood. This rise is the dominant departure from greenhouse gas scenarios that limit global heating to less than 2°C. Thus a comprehensive understanding of methane sources and sinks, their trends and inter-annual variations are becoming more urgent. Efforts to quantify both sources and sinks and understand latitudinal and seasonal variations will improve our understanding of the methane cycle and its anthropogenic component. Nationally declared emissions inventories under the UN Framework Convention on Climate Change (UNFCCC) and promised contributions to emissions reductions under the UNFCCC Paris Agreement need to be verified independently by top-down observation. Furthermore, indirect effects on natural emissions, such as changes in aquatic ecosystems, also need to be quantified. Nitrous oxide is even more poorly understood. Despite this, options for mitigating methane and nitrous oxide emissions are improving rapidly, both in cutting emissions from gas, oil and coal extraction and use, and also from agricultural and waste sources. Reductions in methane and nitrous oxide emission are arguably among the most attractive immediate options for climate action.

This article is part of a discussion meeting issue 'Rising methane: is warming feeding warming? (part 1)'.

## 1. Introduction

**Figure 1.**
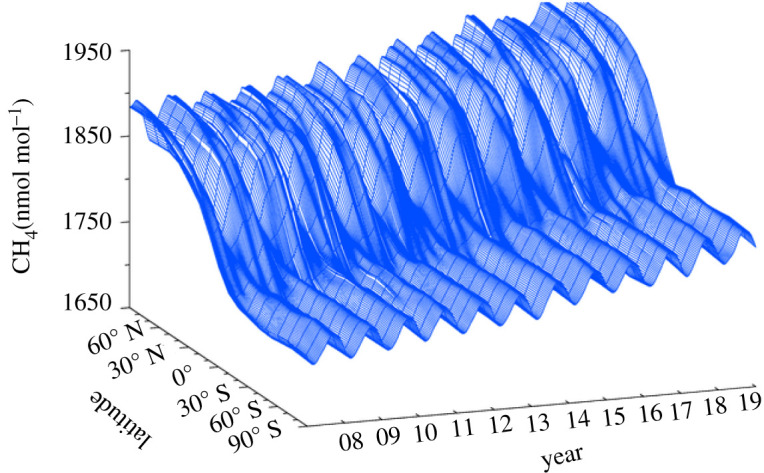
Record of zonally averaged methane in the marine boundary layer, 2008–2019, from NOAA's Cooperative Global Air Sampling Network. Surface shows atmospheric methane as a function of latitude (10° spacing) and time (48 steps per year). Rear of plot: North Pole. Front of plot: South Pole. Note renewed growth from 2008, increasing in 2014. E. Dlugokencky, NOAA. (Online version in colour.)

**Figure 2.**
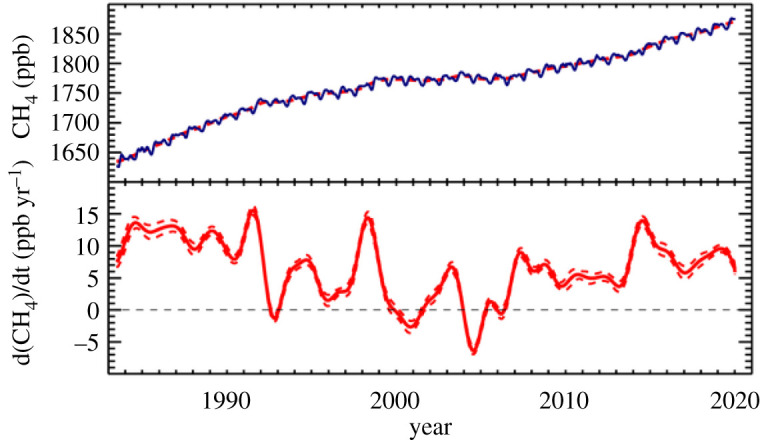
Upper panel: Global monthly mean methane record from the NOAA collaborative global network. Lower panel: Annual growth rates in global methane: note strong growth in the 1980s, reduced growth in many years in the 1990s, stabilization 1999–2006 and renewed growth from 2007. Plots show data to 1 January 2020; more recent data show very strong growth. (Online version in colour.)

Methane is a major climate forcing anthropogenic trace gas, central to our efforts to mitigate climate warming [1,2]. After reaching steady state in 1999–2006, methane's atmospheric burden began rising strongly from 2007 (figures 1 and 2). Growth accelerated in 2014 and again in 2018 and 2019 (figure 2). Preliminary evaluation indicates exceptionally strong rise in 2020, with an initial estimate of greater than 15 ppb growth (https://gml.noaa.gov/ccgg/trends_ch4/). This is an initial estimate likely to be adjusted as further data are acquired, as seasonality means that annual global growth trend estimates cannot be firmly established until some months into the following year. Nevertheless, methane's increase through 2020 (e.g. January 2020 1873.4 ppb; January 2021 1893.4 ppb) is clearly comparable to the highest annual rise in the detailed observational record.

The geographical regions leading the post-2007 rise appear to be primarily in the tropics and low latitude temperate north, although in some years rapid growth has been global. Concurrent with the post-2007 rise, methane's carbon stable isotope ratio has shifted to lighter, more ^13^C-depleted values.

Questions abound: Why is methane rising so fast? Are source emissions rising, or sinks falling, decreasing the magnitude of removal processes, or both? To what extent is this directly driven by anthropogenic sources? Or are climate warming feedbacks also occurring, especially in the tropics and Arctic, for example as wetlands warm or cows flourish in future warmer wetter moist tropics?

The causes driving methane's recent rise are not fully understood [3], but much of the recent rise seems to stem from tropical and warm temperate biological sources, such as cattle, wetlands and landfills [4]. Recent carbon-14 isotopic work suggests that geological emissions are smaller than hitherto thought [5], and that fossil fuel inputs are larger than used in most source budgets [6]. The major sinks, destruction by atmospheric hydroxyl (OH), chlorine atoms (Cl) and oxidation by bacteria in soils, may also be changing [7,8].

The increase in methane's effective radiative forcing from 1750 to 2014 is substantial and has been recently estimated as 0.67 ± 0.17 W m^−2^ [9–11] with a total forcing of about 1 W m^−2^ since 1750 if indirect impacts are included, such as increased NO*_x_* and effects on [OH] [12]. Methane contributes to about a third of tropospheric O_3_ [13], which makes the largest indirect contribution, and methane also contributes to stratospheric water vapour. Thus methane's forcing may be nearly half the 2.1 W m^−2^ impact of anthropogenic CO_2_ in 2020 [14]. Overall, methane is responsible for a significant fraction of the total post-1750 forcing due to all well mixed anthropogenic greenhouse gases.

Monitoring laboratories worldwide maintain the key *in situ* measurements of methane [15]. This gives a potent geographical and seasonal record, to track CH_4_ from pole to pole at large spatial scales, and to assess inter-annual variation as well as long-term growth. *In situ* measurements from stations are supplemented by measurements from aircraft [16] and ships [17]. Satellite retrievals [18–23] give total column pictures, but accuracy in source estimates is difficult to achieve, with averaging kernels peaking in the mid-troposphere, and inferences about emission budgets have wide errors. Zeng *et al*. [24] note that inferring causal linkage between methane observations from space and local surface emissions requires retrievals to be coupled to chemical transport models that cover long-range atmospheric transport. Moreover, currently useful isotopic retrieval to identify methane sources is not currently possible from satellite observation.

Methane has a marked pole-to-pole gradient, with a sharp discontinuity over the inter-tropical convergence zone. This inter-hemispheric contrast was smaller in glacial time [25] and today reflects the dominance of major anthropogenically influenced emissions in the land masses of the Northern Hemisphere. There is strong inter-annual variation, and it is not clear to what extent inter-annual variations are driven by source and sink fluctuations linked to climatic cycles like ENSO, or are primarily driven by inter-annual variations in anthropogenic or natural source activity (which themselves can be influenced by the ENSO cycle).

## 2. The debate about sources and sinks

Budgets of national, regional and global emissions remain uncertain, with major gaps between top-down and bottom-up estimates [26–29]. ‘Top-down’ calculations use measurements of methane and its isotopes in the air and understanding of the known sinks to estimate emissions. ‘Bottom-up’ assessments sum the sources, counting gas and coal leaks, cow breaths, landfill emanations, swamp emissions and the like, but do not demand isotopic balance—for example, volumes of gas consumed multiplied by leakage factors, the number of cows multiplied by nominal emissions per cow, tonnes of coal consumed multiplied by emission rates, etc.

It is not simple to characterize emissions by type. Natural emissions in Holocene time included wetlands, wild ruminants and lightning-lit fires. Anthropogenic emissions include fossil fuel emissions from the gas and coal industry, and thermogenic methane from human-lit fires, as well as biological emissions from landfills, cattle and rice. But farmed cattle have in many areas replaced wild bison, buffalo and antelopes or occupied drained wetlands. Moreover, human-induced climate change is warming wetlands and adding fertilizer run-off, thus increasing natural emissions [30]. It is possible that wetlands may drive much of the methane-climate feedback up to the year 2100 [31]. How should these complex factors be categorized and quantified?

Top-down source assessment, for example by inverse modelling, is aided by the geographical and seasonal information that comes from globally *distributed in situ* measurement time series, and by isotopic tracking [3,23,32]. Regional budgets vary strongly depending on latitude (e.g. the contrast between industrialized northern nations, and the wetland and cow-rich tropics [33]). Tropical emissions are still poorly constrained, due to the scarcity of measurement data, especially for methane isotopes, but likely play a central role in driving recent growth [4,32]. Natural emissions are likely rising both in the rapidly changing moist tropics, and in the northern latitudes with thermokarst development and loss of permafrost. It is not clear how much of the growth is from increasing ruminant populations [34,35] and how much from warming wetter wetlands with increasing paludification [36,37], or from intensifying agriculture and rapidly growing urban and industrial processes. Tropical air pollution is also significant (e.g. [38]), both from fires and from industrialization, with implications for the atmospheric chemistry of methane, especially as much methane destruction occurs in the tropical mid-troposphere.

There is little evidence for a dramatic rise in North American emissions driven by fracking [39,40] and other evidence implicates rising emissions from tropical biogenic sources such as cattle and wetlands [4,20,41]. Arctic emissions growth in the 2009–2019 period may have been moderate despite much concern [42]; see also [43], but recently in 2020 the Inter-polar difference increased significantly, reaching levels comparable to those around 1990, before the economic collapse in the former Soviet Union. However, it is not clear what are the relative impacts on the Arctic of a warm/wet year and methane transport in southerly winds from boreal regions. Global ocean emissions remain uncertain but are probably small, around 6–12 Tg yr^−1^ [44]. Overall, anthropogenically influenced emissions are growing, particularly from agriculture, landfills and waste [45], and may dominate growth.

Methane's sinks may also be changing in response to climate change and air pollution—a decline in methane destruction could explain part of the rise in the burden. The main sink is reaction with hydroxyl radicals, [OH], which acts as the guardian of the atmosphere because of its cleansing detergent role in attacking and oxidizing reactive pollutant species. However, [46] find that decreasing [OH] cannot explain the post-2006 global CH4 increase since the modelled methane burden does not track the observed decrease in global mean *δ*^13^C_CH4_.

Current evidence suggests [OH] is not undergoing dramatic step-change at a global scale, but small global trends and more significant ongoing regional changes in methane lifetime may indeed be taking place [7,47–50]. Trends for an [OH] increase of 2% per decade [47] require a similar increase in sources to reproduce observed concentrations and this is small relative to other uncertainties.

Methane's reaction with [Cl] is a minor sink but has strong isotopic leverage [46]. This has been recently reappraised [51,27,28]. Strode *et al*. [52] found that changing the tropospheric [Cl] field leads approximately to a 0.5‰ increase in *δ*^13^CH_4_ for each per cent increase in how much CH_4_ is oxidized by [Cl]. This large leverage on *δ*^13^CH_4_ points to the need for better evaluation of the impact of the [Cl] sink.

Soil methanotrophy is a third sink [43,53], as yet poorly quantified, with little known about its response to climate warming. Methane uptake by the soil varies with moisture and temperature, so shifts in seasonal cycles and in longitudinal distribution of the soil sink may have impact, particularly with extreme drought and excess precipitation events on forest soils, both in the tropics and north.

Net upward loss of methane to the stratosphere is also not well constrained. In the brightly lit lower stratosphere, methane destruction by [OH] and [Cl] takes place. This is important in the isotopic budget, as air enriched in ^12^C methane by the biological emissions of the tropics rises into the stratosphere above the Inter-Tropical Convergence. The air then moves through the Brewer–Dobson circulation, and returns near the poles, descending to the troposphere via the polar vortex. Here, the remaining methane in the methane-poor descending air is ^13^C rich.

## 3. Questions posed by methane's recent growth

Is the change in methane's behaviour in 2007 dominantly caused by human activity, or by climate change feedback responses, or by both drivers? Anthropogenically caused emissions from both agriculture and fossil fuel use were clearly increasing until the onset of the Covid pandemic [54], while Chandra *et al.* [55] point to a mix of increases in emissions from coal mining, mainly in China, and also the intensification of ruminant farming in tropical regions. Tropical wetland emissions, which are isotopically and geographically hard to distinguish from ruminant emissions, may also be increasing [4,32], and there is a significant risk that emissions will grow strongly with warming [56].

Key questions include the size of the fossil fuel sources (the emissions that are most amenable to mitigation), the relative proportion of tropical emissions from ruminants versus wetlands, and the possible existence of emission pulses, for example from major biomass burning events, huge unrecorded fossil fuel industry leaks (e.g. [18,19]) and Arctic permafrost thaw [37]. There is also poor quantification of the global termite and ocean plankton sources, for which estimates have been little updated for some decades.

The largest knowledge gap is in the discrepancy between the ‘top-down’ and ‘bottom-up’ budgets. The results differ radically, only barely within error margins: Saunois *et al*. [29] find a top-down sum of about 600 Tg for annual emissions, compared to the bottom-up source estimate of about 750 Tg (range about 600–900 Tg). Moreover, the bottom-up estimates imply an annual imbalance of about 120 Tg, hard to reconcile with top-down measurements of annual growth around 5–10 ppb yr^−1^ (1 ppb, globally averaged, is about 2.77 Tg—[4]).

## 4. Use of isotopes

Isotopes provide powerful constraints in assessing global and regional methane budgets, the keys that may unlock full budget quantification and verification. In particular, bottom-up budgets need to tally with top-down isotopic measurements. The three most accessible sources of information are *δ*^13^C_CH4_, *δ*^2^H_CH4_ and ^14^C abundances. In the distant future, polyisotopic time series (e.g. *δ*^13^C^1^H_3_^2^H; [57] may help, but currently it is difficult even to hope for finance to add better *δ*^2^H_CH4_ coverage to the present very sparse global dataset.

**Figure 3.**
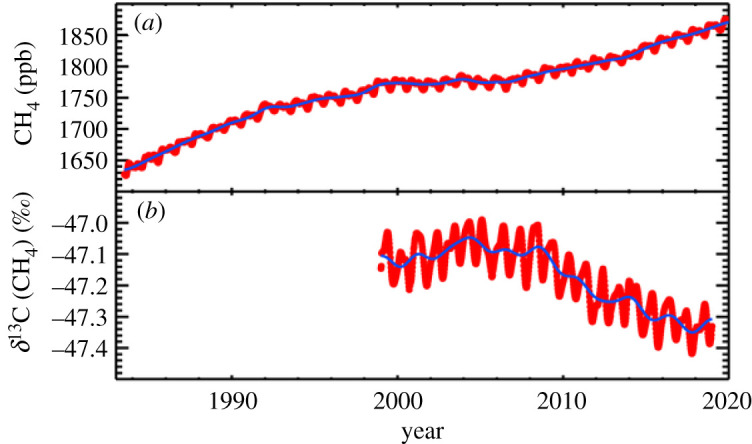
Coincidence of start of onset of global growth in the methane burden with the sustained shift towards more negative *δ*^13^C_CH4_. Red symbols show weekly global means; blue lines show deseasonalized trends. (*a*) Global mean atmospheric methane (as in figure 2). (*b*) *δ*^13^C isotopic ratio in atmospheric methane: note synchroneity of negative shift with start of 2007 rise in the upper panel. University of Colorado, INSTAAR and NOAA data. E. Dlugokencky. (Online version in colour.)

The recent growth in methane has been accompanied by a concurrent isotopic trend in the global methane burden, now sustained for well over a decade, towards more negative *δ*^13^CH_4_. For two centuries prior to 2007, during the 1750–2007 period of rising methane, *δ*^13^C_CH4_ had grown steadily more positive due to anthropogenic emissions from anthracitic coal use, oil and gas, and fires. But since 2007, concurrently with the sharp recent rise in the methane burden (figure 2), atmospheric *δ*^13^C_CH4_ has steadily been shifting negative (figure 3) [4,58]. The data are currently insufficient to determine any global trend in *δ*^2^H_CH4_, although published results [32] show a 2007–2010 decrease in the Arctic (Ny Alesund, Svalbard) and in Antarctica a strong positive trend that had been sustained since 1995 ended in 2007, followed by levelling off until a slight positive shift in 2014.

The causes of the trends remain controversial, with many possible explanations. The possibilities are either (i) a weakening global sink or (ii) increased emissions from methane sources or (iii) changes in both sources and sinks [4,59–61]. The simplest (but not only) explanation of the isotopic shift is that it is dominated by an increasing proportion of emissions from biogenic sources [4,35,62].

Globally, methane's mass and different isotopic budgets must all balance, with inputs equal to removals plus increase in the atmospheric inventory. Sources and sinks have a wide range of impacts on the global budget for *δ*^13^C_CH4_ [32,63,64] (table 1). Quantification of the global methane budget must satisfy the isotopic algebra, but to date, important global budget assessments [29,30] have not been able to use isotopic constraints.

**Table 1.** Approximate isotopic source signatures of the major inputs to the global budget, and the fractionations imposed by the major sinks. Note the caveat that the variability in each source is likely greater than the s.d. shown here.

source	*δ*^13^C_CH4_ (‰) (±2 s.d.)^a^	*δ*^2^H_CH4_ (‰) (±2 s.d.)^a^	flux (Tg yr^−1^)^b^
microbial	−62 (±12)	−317 (±66)	516 (360–463)
fossil	−45 (±21)	−197 (±102)	155 (112–194)
biomass burning	−26 (±10)	−211 (±30)	31 (26–46)
sink	^13C^*ε* (‰)	^D^*ε* (‰)	
[OH]	−3.9^c^, −5.4^d^	−145^e^, −200^f^, −227^c^ −237^g^	553 (476–677)
soil uptake	−16 to −26^h,i,j,k^	−90^k^, −62^k^	30 (11–49)
tropospheric [Cl]	−62^l,m^	−337^n^	11 (1–35)
stratosphere	−3^o^	—	31 (12–37)

Source signatures and fluxes (with ranges).

^a^Sherwood *et al*. [64].

^b^Saunois *et al*. [29]. Sink fluxes (with ranges) and isotopic fractionation factors (where *ε* is defined as the ratio of the reaction rate constants minus 1, with the rate constant for the more abundant isotopologue as the denominator).

^c^Saueressig *et al*. [65] (296 K).

^d^Cantrell *et al*. [66] (273–353 K).

^e^DeMore [67] (298 K).

^f^Gierczak *et al*. [68] (298 K).

^g^Joelsson *et al*. [69] (298 K).

^h^King *et al*. [70].

^i^Tyler *et al*. [71].

^j^Reeburgh *et al*. [72].

^k^Snover *et al*. [73].

^l^Saueressig *et al*. [74] (297 K).

^m^Crowley *et al*. [75] (298 K).

^n^Saueressig *et al*. [76] (296 K).

^o^Lassey *et al*. [77].

Globally, regionally and locally, mass balance and isotopic balance apply: inputs must equal removals and outward fluxes. Each source has major latitudinal and seasonal variation across the planet, and sinks are similarly variable with season and latitude. Modelling needs to take this seasonal and latitudinal variability into account. Successful resolution of the global budget must account for both for geographical differences and inter-annual variability in the seasonal cycles for *δ*^13^C_CH4_, but this is hard to resolve given the underinvestment in long-term *in situ* time series for isotopic data.

Table 1 compiles approximate isotopic source signatures for major inputs to the global budget. Source inputs and sink impacts balance to give the global atmospheric mix. But source signature information, especially for *δ*^2^H_CH4_ remains sparse. Moreover, sources vary greatly, both by latitude for natural sources and locally for anthropogenic emissions. To take one example, the isotopic impact of coal production is complex: methane from anthracitic coals (e.g. used in steel making) tends to be ^13^C rich but methane from brown coals and lignite used in power generation can be more depleted than atmospheric methane [78].

Figure 3 compares changes in the mixing ratio and the *δ*^13^C_CH4_ record. The global methane burden has a *δ*^13^C_CH4_ value somewhat more negative than −47‰, which reflects the impact of a bulk global source around −53‰, and a sink-imposed shift of about 6‰, dependent on the relative weightings of the different sinks. For *δ*^2^H_CH4_, the combined sink-imposed shift is much larger, resulting in a *δ*^2^H_CH4_ composition of the global burden around −90‰ that is significantly more enriched in ^2^H than the bulk signature of the sources.

**Figure 4.**
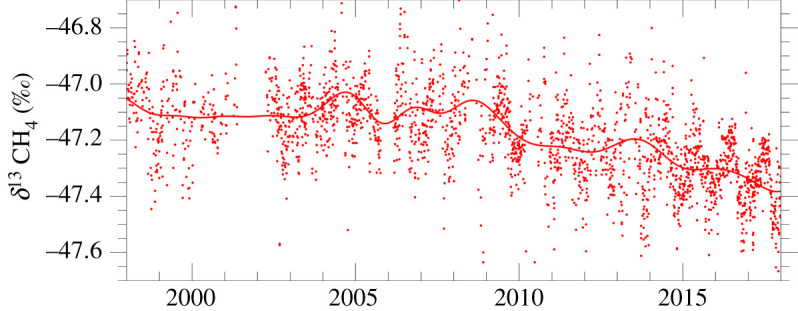
*δ*^13^C_CH4_ record from weekly air samples collected at Mauna Loa Observatory, 1998–2017. Note sustained shift to more negative values since 2007. Later data are as yet unvalidated. S.E. Michel. (Online version in colour.)

The extent of the seasonality of the changing inputs and inter-annual variation means that isotopic equilibration is never achieved [4]. Figure 4 shows point-by-point data from one station, Mauna Loa, Hawaii, to illustrate the isotopic variability in different airmasses. If the source isotopic signature changes, for example from a major input pulse, the global atmospheric value will also shift, but with a lag that can take decades [79]. Thus the addition of a sharp pulse of new methane from thermogenic fossil fuels (*δ*^13^C_CH4_ more positive than −47‰) or wetlands (much more negative) will shift the global value for some years, before the sinks bring the isotopic ratio closer to equilibrium.

**Figure 5.**
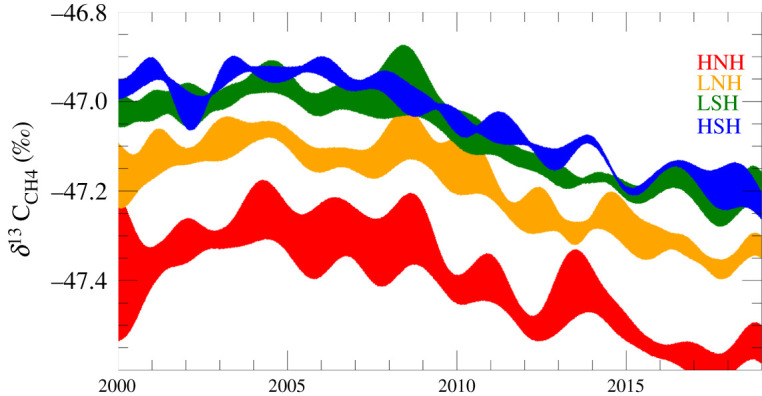
Zonally averaged *δ*^13^C_CH4_ and its uncertainty range for high latitude Northern Hemisphere (HNH), low latitude Northern Hemisphere (LNH), low latitude Southern Hemisphere (LSH) and high latitude Southern Hemisphere (HSH). These are semi-hemispheres: equator-30° = LNH/LSH and 30–90° = HNH/HSH. For practical purposes, they represent equal volumes of atmosphere, approximately 25% of the global total, each. Uncertainty estimates consider atmospheric variability, analytical uncertainty and the uncertainty of the sampling network. S.E. Michel. (Online version in colour.)

There is regional and latitudinal information in the isotopic record (figure 5). Since 2007, the negative *δ*^13^C_CH4_ shift has been sustained across all semi-hemispheres, while the total methane burden has been growing rapidly. A first guess explanation of these changes is that biological sources have grown, causing negative *δ*^13^C_CH4_ shifts in the temperate Northern Hemisphere in 2007 but since then especially in the tropics and Southern Hemisphere. However, note that these semi-hemispheric trends come from a very small network of stations.

Schwietzke *et al*. [6] used isotopic evidence to find that methane emissions from fossil fuel industries are much higher than hitherto estimated. Similarly, Lassey *et al.* [77] explicitly showed bottom-up compilations were inconsistent with 15 years of Δ^14^CH_4_ data from both Antarctic firn air and New Zealand air samples. Methane's historic record is held in ice cores, which are revealing surprises: the late glacial and pre-industrial records suggest that geological and other ancient carbon emissions were small [5,80,81]. If this inference is correct, that in turn suggests that nearly all of the fossil emissions observed by Schwietzke *et al*. [6] are anthropogenic and thus increases our estimates of modern fossil fuel emissions, and confidence in constraints on them from 30 years of Southern Hemisphere Δ^14^CH_4_ data [82]. However, there are alternative explanations, such as emissions from old carbon sources in peat [83], changes in sinks [84], and Fujita *et al*. [32] observed that global fluxes obtained by inverse modelling could overestimate ‘prior’ biogenic emissions and thus overestimate the proportion of biogenic sources and underestimate fossil fuel and biomass burning.

The problem remains unresolved [4]. The most critical gap in data is arguably the need for multiple high-quality *in situ* measurement time series, including not only measurement of methane's mole fraction but also more *δ*^13^C_CH4_ and reinstated *δ*^2^H_CH4_ time series, from multiple stations, especially in the tropics, where data are very sparse.

## 5. Emission verification

‘Trust but verify’ was the basis of nuclear weapons treaties: arguably the success of the Nuclear Test Ban came because the negotiators were careful to implement independent external verification by the Comprehensive Nuclear Test Ban Treaty Organisation (CNTBTO). This was critical to the success of the Treaty. For the Paris Agreement to succeed, there is a strong argument that the United Nations Framework Convention on Climate Change (UNFCCC) needs similar verification of national methane emissions declarations, especially in the tropics where data are very sparse.

But verification is lacking. Compliance with treaty commitments is self-declared by nations. The Paris Agreement and the UNFCCC are self-monitored. Although there are strong parallels between the global Nuclear Test Ban Treaty and the legally binding Paris Agreement treaty on climate change, there is currently no equivalent to CNTBTO for tracking the comparable global commitment to reduce climate-forcing emissions.

The United Nations’ World Meteorological Organisation's Global Atmosphere Watch (GAW) is the closest analogue. This is a collaborative partnership of science teams. Through its participants, GAW does maintain SI-traceable standards to calibrate analyser response and ensure data are comparable across international measurement networks. But sustaining monitoring programmes is very challenging, and also unrewarding in a funding environment dominated by hypothesis-testing priorities and publication success. For many sites maintained by university-based scientific teams, there is a problem of ‘immediacy’—funded by a chaotic succession of short-term grants demanding rapid impacts and publication, long-term observation is extremely difficult to sustain. Thus long-term funding commitments are absent in most countries. Sustained calibration and the task of ongoing inter-comparison between different laboratories and networks, necessary to maintain global perspective, is especially difficult [85,86].

In this context the multi-decadal commitment of US NOAA's Global Greenhouse Gas Reference Network is critical: it is the solid basis for the bulk of our global understanding. Led by NOAA, our networks have defined the scale of the issues, provided a baseline understanding and give insight to future network and assessment design. But Nisbet *et al*. [4] pointed out that unfortunately, with limited measurement datasets, it is not currently possible to be definitive about global and regional budgets. The paucity of the isotopic record, from so few stations, does demonstrate the urgency of gathering more information, both in monitoring the changing atmosphere, and also in testing global and regional budgets and verifying emissions.

Large discrepancies remain between bottom-up inventory-based assessments and top-down measurements [29]. In many cases, inventory assessments of leakage may be far from what is actually measured [87,88], a problem that may in future be rectified as direct top-down measurement of emissions is increasingly feasible (e.g. [27,28,89,90]).

Satellites are helpful but quantitative and effective verification needs the detailed and isotopic information provided by *in situ* data. The value of *in situ* measurement has been shown by Bergamaschi *et al*. [91], who found total CH_4_ emissions of about 27 Tg for the then-28 states of the European Union, which contrasted with the total anthropogenic CH_4_ emissions reported to the United Nations Framework Convention on Climate Change, of 21 Tg in 2006 and 19 Tg in 2012.

## 6. Measurement needs

Several points are clear:

(a) There is strong need for better, more sustained long-term *in situ* measurement time series of methane and its isotopes, in both northern and southern Tropics, including in remote locations in the continental interiors. This is not expensive and currently the USA, Australia, New Zealand and South Africa are carrying a disproportionate share of the burden of Southern Hemisphere observations. New sampling techniques, for example sampling air in the free troposphere using UAVs [92] could contribute much to better understanding of the air in tropical continental interiors.
(b) Bottom-up emissions inventory budgets need to be isotopically balanced, tested for compatibility with methane actually emitted to the air, to differentiate between source inputs. Locally and nationally, emissions declarations (e.g. to UNFCCC) need to be tested against isotopic ratios measured in plumes and air masses. On a global scale, the bottom-up isotopic budgets need to be constrained by the time-evolving top-down measurements.
(c) Top-down assessments of emissions, locally, regionally and globally, must similarly be tested against the isotopic constraints. In particular, there is need to gain better quantification of the impacts of the variability of [OH] and [Cl] on the global CH_4_ budget.
(d) Dedicated flux and isotopic measurement campaigns are needed, especially in the tropics, to improve understanding of source signatures, to help strengthen tropical national emission inventories, which are currently rudimentary, and to search for climate warming ‘unknown unknowns’.
(e) Currently. measurement priorities are primarily focused on mole fraction and *δ*^13^C_CH4_, but the information is inadequate to constrain global budget inversions. Measurement of *δ*^2^H_CH4_ need to be added to the portfolio of time series measurements, tracking *δ*^2^H_CH4_ as it evolves globally, and with much better determination of *δ*^2^H_CH4_ isotopic signatures of sources and sink fractionation. Because fractionation of hydrogen isotopes in the reaction with [OH] imposes seasonal and latitudinal gradients on *δ*^2^H_CH4_ this may be useful for constraining the [OH] sink.
(f) More generally, networks and assessment technologies need to be designed to be capable of resolving UNFCCC commitments and policy impacts; thus providing, like CNTBTO, a wholly independent pathway to trust, but also to verify, reported emissions inventories.

## 7. Can mitigation succeed in cutting emissions to Net Zero?

Can we bring methane under control? Mitigation options are rapidly improving, and there is the impetus to reduce our dependence on coal and natural gas. There have been significant recent advances in measurement instruments and the scope for their deployment [93]. This permits better identification and quantification of major anthropogenic sources, facilitating emission reduction. In particular, mitigating gas vents and leaks, and capturing manure and landfill emissions from tropical megacities is increasingly attractive.

Methane potentially provides many good near-future (this decade) mitigation targets. Cutting methane emission is broadly cost-effective compared to methane removal from ambient air [94], though with appropriate technology in appropriate high methane settings, removal may indeed be an option [95,96]. Jackson *et al*. [97] point in particular to the need to more research into removal methods.

For example, in local settings where elevated methane is habitually present in the air (e.g. in cattle barns, or active faces of landfills, removal may be feasible [93]. There are many attractive targets such as cutting emissions from fossil gas use, decarbonizing heating and cooling systems, ending coal mining and burning, reducing landfill and biodigester emissions, and by improving agricultural practices both in temperate and tropical farming [93].

To address these targets in more detail, cutting leaks and deliberate venting from natural gas production sites is an obvious immediate target, and is among the least costly ways to cut greenhouse emissions. Waste is widespread. For example, Lyon *et al*. [98] point to insufficient infrastructure capacity for handling and delivering gas. They suggest the Permian Basin, one of the largest oil and gas producers in the USA, is in a state of overcapacity in which rapid growth in gas production can exceed midstream capacity, leading to high methane emissions. The gas industry has abundant super-emitters, and also emissions from abandoned wells [99]. Ground, airborne and rapidly improving satellite leak-detection and mapping can all be used to identify emitters [100–103].

Decarbonizing heating, transport and electricity generation is critical to meeting Net Zero goals. More generally, it is time to remove all fossil fuels from the global economy [104]. In rebuilding the energy infrastructure, ending methane emission is an obvious first option. Fugitive gas is not only from pipes: there are significant emissions also from domestic boiler exhausts and from installations like gas governors and offtake stations, which can be also readily mitigated [93]. Simple emission reductions in the gas transmission network can make a significant, inexpensive initial contribution.

In the medium term, there is wide scope in developed nations for shifting fully to electric or solar heating, ending low-pressure gas distribution, perhaps reusing urban local gas pipes for power wires and communication fibres. However, although renewable green hydrogen may be enticing, moves to introduce hydrogen to the domestic supply need careful thought, given the possible impacts of hydrogen leaks on air pollution, on stratospheric ozone and as an indirect greenhouse gas [105]: electrification of heating may be preferable.

Emissions from the global coal industry, during mining, pulverization and use, are poorly quantified but may be very large, around a third of total fossil fuel emissions [29]. Growing Chinese coal emissions may have been a significant supplementary contributor to the recent rise in methane [106]. Coal is increasingly becoming uneconomic as a source of electricity and new renewable plants are becoming more cost-effective than new coal-fired power plants [107], but it may be many decades before this leads to full shut-down of coal power in coal-dependent India, South Africa and China (all of which are very vulnerable to climate change). Coal-sourced methane (and CO_2_) emissions from steel making are globally significant. These can be eliminated if renewable ‘green’ hydrogen is used as an alternative reductant. If so, hydrogen leaks will need careful attention (see above, and Warwick *et al*. [105]).

There are some clearly egregious methane-emitting excesses in the carbon economy. Wealth stores are examples. Consider mining coal to produce electricity to power Bitcoin mining. This emits CO_2_ and methane, imperilling the climate to create a source of wealth that is wholly abstract. One cannot eat a Bitcoin. Gold mining, that older source of wealth, is also highly energy-demanding but at least the gold is then permanently recycled in the economy, useful eventually for many purposes: electronics, jewellery or even in drugs. In parts of tropical Africa, such as South Sudan, cattle are currency [108]: they are used primarily as wealth stores rather than food. Replacing cattle with formal currency (for example via mobile phone banking, increasingly popular in Africa) brings greenhouse gas benefits and may reduce over-stocking and habitat destruction.

Landfills are a very attractive target for mitigation of methane emissions, increasingly so in the African and American tropics and in southern Asia. Saunois *et al*. [29] estimate global waste emissions as about 65 Tg CH_4 _yr^−1^ or about 12% of total global anthropogenic emissions. In the UK, through strong legislation, waste sorting and composting, and careful landfill remediation, methane emissions from landfills have decreased by 76% between 1990 and 2018, brought about at relatively low cost and with little political debate, though in the UK methane leaks from biodigesters can be large and need to be controlled [109]. Despite this success, there is more to do as landfill emissions still constitute 28% of total UK methane emissions [110]. Few other nations have been as successful.

For many tropical megacities, waste is dumped in little-sorted, often unmanaged heaps, often on fire. Yet emission reduction is neither difficult nor costly, nor does it demand high skills: this is not high-tech mitigation, but simple good sense. Waste sorting is cheap, to remove and compost organic matter such as waste food. Composting can be both beneficial to local small scale agriculture and also cut the use of N_2_O-releasing fertilizers. Biogas production from organic matter can be profitable in cities far from gas sources, Waste piles can be piped to extract gas. Methanotroph-hosting soil cover is a very inexpensive way of reducing methane emissions and other gas hazards [53,93,111]. Yet in India, the overwhelming bulk of waste is put into open dumps without further treatment [112]. Widely for many tropical megacities, landfill soil cover can be thin, slow or wholly lacking.

Harmsen *et al*. [113] show that strong attention must be paid to tackling agricultural emissions, both in temperate and tropical agriculture [114,115]. Ku-Vera *et al*. [116] have shown that in cattle grazing low-quality tropical forages it should be possible to find ways to mitigate enteric CH_4_ emission yet effectively increase efficiency and productivity. In developed nations, rapid and inexpensive reduction of emissions should be possible with better aeration of anaerobic agricultural manure pools.

Smith *et al.* [117] make the strong point that moving half of human nutrition to vegetarian diets and reducing food waste by half might reduce methane emissions by around greater than 50 Tg CH_4 _yr^−1^. Reducing ‘industrial’ dairy farming, with its manure tanks, by switching to more expensive milk from ‘organic’ dairy cattle would likely cut emissions but may not be popular. In agriculture, methane is not the only emission: apart from CO_2_ from energy use, N_2_O is a major emission (see §8 below). There is a danger that reducing emissions of one gas (e.g. methane) may increase emissions of another (e.g. N_2_O), or increase demand for crops from tropical ex-forest land. Thus mitigation actions should address all three gases together.

Chiri *et al*. [118], studying termite mounds, have shown the efficacy of methanotrophy (methane consumption by aerobic methane-oxidizing bacteria) in locations where the air's methane content is intermediate (0–100 ppm) between the high amounts encountered by landfill soil methanotrophs and in cattle barns, and the low methane contents of air that is the substrate for forest soil methanotrophy. Termite mounds mitigate between 20% and 80% of their emissions [118]. The implication is that the use of bacterial methanotrophy to reduce methane emissions is feasible around landfills and farming facilities where intermediate methane contents are present in the air.

Methane mitigation is feasible everywhere, but particularly in cutting fossil fuel, landfill and crop waste emissions. China, India, Brazil, the USA, Australia and South Africa, all of which are large emitters from these sources, are all vulnerable to climate change. Mitigation of anthropogenic CH_4_ emissions can potentially offset CO_2_ emissions around 200 Gt of carbon [119]. By 2050, Höglund-Isaksson *et al*. [120] consider it technically feasible to reduce emissions by 40% compared to 2015 amounts, but expect rather less in practical terms. However, note that this pessimistic analysis was based on socio-economic modelling, not on analysis of likely scientific and technical developments: the likely future of a model world without true innovation [121,122].

## 8. Policies for methane mitigation

**Figure 6.**
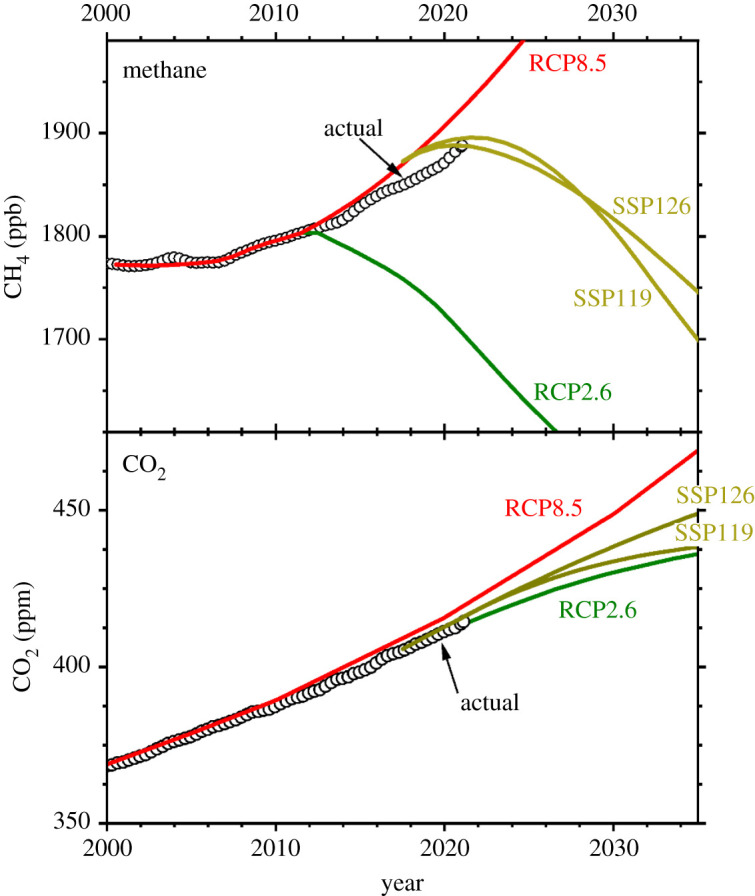
Top panel shows the evolution of methane's mean global atmospheric mixing ratio (green open circles) compared to Representative Concentration Pathways RCP2.6 and RCP8.5 [123] and shared socio-economic pathways (SSP) from [124]. RCP2.6 scenario, which is consistent with the Paris Agreement, peaks in 2012, whereas SSPs peak in the early 2020s, and RCP 8.5 (dotted) continues to rise throughout the twenty-first century. Lower panel shows similar evolution scenarios for CO_2_. Radiative forcing used here is from Etminan *et al*. [11] and the figure is updated from that in [4] using NOAA/GML mole fraction data to January 2021. M. Manning. (Online version in colour.)

To attain Net Zero by 2050, reducing atmospheric methane to pathways envisaged in scenarios compliant with the UNFCCC Paris Agreement (figure 6) will have rapid impact on climate warming [125]. By contrast to CO_2_, for which a proportion of emissions remains in the air for centuries, the lifetime of methane is between 9 and 10 years [50,126]. The instantaneous lifetime rates vary strongly with latitude, from less than 2.5 years in the tropics to greater than 20 years in the high latitudes [4]. The so-called ‘perturbation lifetime’—the time it takes in a theoretical model atmosphere for a methane input to decay, is about 12.4 years [12], which is also relevant to mitigation discussions [127].

The recent UNEP CCAC [1] report demonstrates the very significant cost-benefit advantages to cutting methane as part of a wider programme to phase out greenhouse warming due to fossil fuels. To address climate warming globally, the reduction of methane emissions is a key mitigation strategy and must play a major role if a 1.5°C temperature stabilization trajectory is to be achieved [113,128].

Thus methane mitigation is a necessary complement to CO_2_ reduction; but this will need determined international action. But thus far the outcome has not been good (figure 6), even for the simpler and cheaper actions, for example to end gasfield emissions [98]. As a general point, over-long commitment to sunk investment in fossil fuels is likely to be value-destroying as assets such as coal mines and power stations become stranded.

The UK Climate Change Committee's Sixth Carbon Budget [129] advises the UK government on pathways to progress to Net Zero climate warming emissions. The report lists a wide range of suggested measures towards a new economy. There is much scope for improving monitoring systems, which are important during the transition to Net Zero, to detect and prevent methane leaks from the gas network. Many policy debates can be expected, with many competing invested stakeholders, as well as the social and cultural significance in current forms of land-use and agriculture. There are no easy answers.

The whole cycle quantification of climate warming impacts is necessary but not simple and will demand international collaboration. Helpfully, reducing CO_2_ emissions also widely reduces methane emissions from fuels that emit CH_4_ during extraction and distribution. However, food is a more complex challenge. Is it better to cut meat consumption in industrial countries at the price of increasing imports of crops from ex-forest in the tropics? Pasture-sourced food is central to human nutrition in Africa and India. There is not necessarily a net climate forcing advantage in terminating aerobic pasture-fed ruminant products from grazing low-quality land, and replacing the food supply with intensively farmed crops from expanded arable land. The debate is open and the ongoing discussion may test priorities and value systems. Cutting methane brings short-term gain.

In particular, there is much opportunity for methane mitigation in developing countries [130]. Tropical nations themselves are among the nations most vulnerable to climate change and methane-linked air pollution is widespread in the very large new tropical megacities. By contrast to the task of restraining CO_2_ emissions, developing nations share responsibility for addressing the methane budget as they are major emitters and should thus be major contributors to reduction efforts, for example in landfill management [93]. Through technology transfer and capacity-building, aid could be focused on methane mitigation [131]. But methane mitigation is not difficult or expensive: the primary responsibility for mitigation is for these nations themselves.

Anthropogenic emissions of atmospheric methane also have major implications for human health, as emphasized by the recent UN Environment Programme Report [1]. In addition to weather-related impacts on human health (e.g. from heatwaves, droughts, storms, floods and the related spread of vector- and water-borne diseases), high methane mole fractions over polluted heavily populated regions lead to the production of ozone, with consequent increases in respiratory morbidity and mortality for humans, and wide impacts on plant life. Mitigating methane emissions thus correspondingly also reduces these impacts on human and plant health.

Thus methane's shorter lifetime and stronger short-term radiative impact than CO_2_ both favour a policy of acting quickly on methane while simultaneously driving the larger, longer-term economic shifts necessary to cut CO_2_ emissions, the essential task of mitigating climate warming. Moreover, cutting methane emissions has very strong public health benefits also, because of methane's role in ozone formation in air pollution [1]. But currently, the evolution of the atmospheric methane burden is far from the hopes of the Paris Agreement. Figure 6 compares scenario pathway RCP 2.6, which was consistent with the Paris goals, with current hopes and the actual record.

## 9. Nitrous oxide (N_2_O)

Nitrous oxide is badly neglected but important, responsible for about 0.2 W m^−2^ of radiative forcing relative to 1750 [12,132] with strong recent increases in forcing. The current atmospheric N_2_O value, above 330 ppb, is sharply higher than the 270 ppb values typical of the years 1000–1800 CE [133] and the atmospheric lifetime is over a century with the global loss rate in the early years of this century about 13 Tg yr^−1^ [134]. Griffis *et al*. [135] pointed to large inter-annual variability in emissions, and the sensitivity of emissions to climate. They suggested emissions will increase substantially with climate warming, in a major challenge to the UN Paris Agreement.

**Figure 7.**
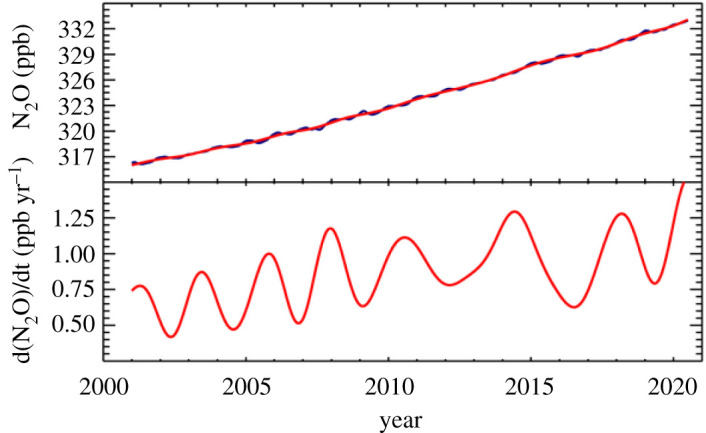
Globally averaged N_2_O, 2001-present. Note sustained growth at increasing rates. NOAA data: E. Dlugokencky. (Online version in colour.)

Growth in atmospheric N_2_O has been sustained and the annual increment is increasing (figure 7). Between 2007 and 2016, global N_2_O emissions were about 17 Tg of nitrogen per year [136]. Although top-down inversions are poorly constrained, especially in Africa, Southeast Asia and southern South America [136] it is likely that N_2_O emissions are growing rapidly, especially in Brazil, China and India, and there is risk of N_2_O–climate feedback [135,136], with warming helping drive emissions. Anthropogenic or human-influenced emissions were over 7 Tg annually, much of which is from the use of nitrogen fertilizer in croplands, with oxidation of ammonia [136,137].

Mitigation of urban emissions is feasible, for example for emissions from wastewater [138], especially from industrial sources (e.g. leaks from nylon manufacture in newly industrialized countries). However, although it may be possible to reduce emissions from wastewater [138] the feasibility of the task may be limited by the cost in energy and effort [139]. A minor source, that may be amenable to control either by taxation or regulation, is from whipped cream chargers and their misuse in small steel cylinders for so-called legal highs. The absence of regulation on these uses imply to the public that N_2_O emissions are unimportant: trivializing the climate implications. Note, however, that nitrous oxide is also a valuable anaesthetic gas, for which purpose its use should be protected.

Mitigation of N_2_O emissions in agriculture [140] is possible. Increased fertilizer use efficiency and reduced N_2_O emissions from agricultural soils could both be achieved by better fertilization strategies [137]. N_2_O production by nitrifier denitrification is promoted in settings low in oxygen, under fluctuating aerobic–anaerobic conditions [141]. Reduction in nitrous oxide emissions is feasible, for example by changing manure spraying and managing soil oxidation levels [142].

However, the task of changing agricultural emissions is complex and may have unexpected knock-on impacts. For example, in India growing rice with intermittent flooding rather than under continuous flooding might reduce methane emissions but Kritee *et al*. [143] suggested that N_2_O emissions from rice across the Indian subcontinent might be 30–45 times higher if rice were grown this way. Such complexities need to be taken into account when discussing increasing crop production to replace of foods derived from methane-generating ruminants (though note that ‘industrial’ ruminant farming also depends heavily on nitrogen fertilizers for fodder and grass).

Replacing fossil fuels with biofuels can lead to an increase in N_2_O sources unless there is also a move away from using ‘first generation’ biofuels such as rapeseed and maize [144]. Biological removal of nitrous oxide from the air, for example around manure slurry, may also be feasible, using one of the three enzymes capable of reducing nitrous oxide: namely nitrogenase; multicopper oxidase and nitrous oxide reductase [145].

To summarize, currently, there is little research and little action on N_2_O. This is a serious gap in global policy. N_2_O is dangerously neglected despite its important and rising contribution to climate forcing.

## 10. Challenges

Much about methane and nitrous oxide remains unknown.

The knowledge gap between the top-down and bottom-up methane budgets [29] needs urgent resolution. This gap challenges the veracity of the inventories painstakingly collected by the UN Framework Convention on Climate Change.

In determining methane emissions, there are three major sources of information: in addition to measurement of methane, careful geographically spread time-series monitoring of methane's C-isotopes and H-isotopes will help identify and locate sources. Mole fraction alone is not enough: to distinguish between, for example, methane from gasfields and methane from cattle where sources are co-located, for example in Texas, isotopes are needed.

Currently, the NOAA Global Greenhouse Gas Reference Network (GGGRN) measures CH_4_ abundance at about 100 sites, but there are few in the tropics. Other nations like South Africa help, but coverage is inadequate and budgets slim. This absence of measurement places significant limitations on the accuracy and value of global modelling. There are no long-term high-quality greenhouse gas time series from moist interior Africa and South America, and very few in moist tropical Asia. *δ*^13^C_CH4_ time series are maintained at a 22-member subset of sites, but methane isotopes in the tropics are only available from the US Pacific island sites and Ascension Is. in the Atlantic. Currently, this thin data stream is not enough to constrain a fully useful solution to the top-down versus bottom-up problem. If the global budget is to be solved, a bigger dataset is likely to be needed, especially with isotopic time series for *δ*^2^H_CH4_ as well as *δ*^13^C_CH4_, and also with more inland sites in the tropics. It should be noted that though *δ*^2^H_CH4_ measurement in the GGGRN stalled due to analytical setbacks, it is hoped it will soon restart.

Isotopes can be used to place powerful constraints on inputs from different sources, such as fossil fuels, fires, wetlands and agriculture into global methane budgets. But to do this, the isotopic source signatures of major emissions need to be known. For fossil fuels, while gasfield methane has been widely measured for *δ*^13^C_CH4_, and to a lesser extent for *δ*^2^H_CH4_, the isotopic source signatures of methane from tropical sources have rarely been measured. There are very few determinations of *δ*^13^C_CH4_, and *δ*^2^H_CH4_ for methane from annual tropical (C4) grass fires. Some tropical wetlands have been studied for their methane isotopic source signatures, but mainly from chambers on the water surface, missing methane that is channelled up stems of tall grasses like papyrus, or up trees. Similarly, methane from tropical cattle has rarely been measured isotopically *in the pasture*.

In particular, emissions from wetlands and ruminants are difficult to differentiate as they often have very similar isotopic signatures, because both come from anaerobic methanogenesis. Where both sources are closely juxtaposed, clues come from geography—where the emissions come from, inferred by back-trajectories, and carbon inputs (e.g. proportions of isotopically ^13^C richer C4 plants like maize or papyrus, or ^12^C richer C3 trees or other grasses).

To determine regional isotopic source signatures for methane from regional-scale tropical sources, low flying aircraft campaigns are needed, to collect air in the boundary layer. With such data, it should become possible to use isotopic and geographical information jointly to address the *natural* versus *anthropogenic* debate, and the allied problem of *cows* versus *wetlands*. However, in many cases this is not possible—many tropical wetlands also have high populations of cattle.

Methane also has poorly quantified other sources where placeholder flux guesses from the 1980s still maintain footholds in computer models of atmospheric chemistry and transport. Examples include fluxes from termites (but see [118]); from geological sources (but see [5]); from shelf seas, especially the Arctic, and from the open ocean (but see [146,147]). Also, soil sinks need further study (but see [43]). Some of these orphan placeholders are being replaced with results from more recent studies (e.g. see [29]).

Removal of methane from the atmosphere is feasible and in certain circumstances worthwhile, even though the lifetime of methane is much shorter than CO_2_. This topic is discussed elsewhere in this volume.

For N_2_O, the problems go deeper. Methane is the Cinderella gas, neglected compared to CO_2_. But nitrous oxide has had even less attention. Cutting N_2_O emission would make a major contribution to mitigating long-term climate warming. Yet discussion of this important gas is almost absent from the political debate.

## 11. Conclusion and outlook

Methane's rise is important and challenging. We still do not understand it, and yet we need to in order to achieve our climate goals. The feedback cycles of methane and its incremental impacts can complicate efforts to slow climate warming. The major needs are for improved *in situ* measurement, including *δ*^2^H_CH4_, the third leg of the information tripod; for better resolution of bottom-up and top-down budgets; for improved understanding of the spatial and temporal patterns of CH_4_ sinks, including how they are changing with time; and for much better measurement and understanding of tropical budgets.

The UNEP CCAC [1] report clearly demonstrated that reducing the global methane burden is one of the most effective strategies to limit global climate warming and to meet the goals of the UN Paris Agreement, as well as to improve health. Hopes are high [122] but practical reality to date is disappointing (figure 6). Targeted methane mitigations are realistically capable of cutting annual methane emissions by 180 Tg, or 45% of anthropogenic inputs. Not only would this greatly help the task of meeting the warming goals of the Paris Agreement, but the ancillary impacts on air quality would bring major benefits for human health and crop production [1].

Though methane's rise challenges us, methane also offers hope [95]. The opportunities for cost-effective mitigation are large and many ways of cutting emissions can be addressed immediately and inexpensively. Carbon accounting for methane should be full cycle—thus biogas generators should include leak assessment, reductions in ruminant farming should include impacts of replacement foods. In the longer term, it is not unrealistic to hope to bring the global atmospheric methane burden down towards its pre-industrial equilibrium. Cutting methane is arguably the most powerful immediate intervention needed in meeting the Paris Agreement target.

As for N_2_O, the policy has failed. There is minimal attention.

## Data accessibility

No primary data are reported.
